# Annual Address

**Published:** 1861-05

**Authors:** A. Means


ATLANTA

### Medical and Surgical Journal.

Vol. VI.] MAY, 1861. [No. 9.

### ORIGINAL COMMUNICATIONS.

### ARTICLE I.

*Annual Address. Delivered before the Medical Association of the State of Georgia, at its session in Atlanta, April 11th, 1861.* By A. Means, M.D., LL.D.

*Gentlemen, Members of the Medical Association:*

Disappointed as you have been, in the delivery of the Annual Address anticipated from the able appointee of this honorable body, whom, together with his distinguished Alternate, we regret to report as providentially absent on this occasion, I have consented, on hasty notice, to substitute, as best I may, the performance of that duty, rather than permit it to go by default. Upon past notes and past reflections, however, I must be permitted liberally to draw, for the staple of the present Address, but as they have never yet been submitted to the public eye, I take the liberty to embody them here, with the diffident hope that, at least, some of the facts and arguments presented may engage the attention of my enlightened auditory, and be assigned an humble rank amongst the *stronger* defenses which have so long environed the citadel of Medicine, and like granite ramparts, repulsed and blunted the malignant shafts of unscrupulous assailants.

The profession which we have chosen, gentlemen, is venerable for years, and illustrious for learning, and may well excite, into bold play, the noblest energies of our intellectual and moral natures. It is no mere fungous growth of fragile texture and ephemeral duration, germinating in the dark, to die in the sun-light—but the sturdy product of successive centuries, whose deep roots and stately trunk have stood the shock of a hundred tempests, and yet bear their green honors luxuriant before the eye of the world. In short, the advocates and cultivators of medicine may pass away by thousands, but its *principles* and its claims, are as fixed as "the everlasting hills," founded in the truth of science, and the necessities of our race, and destined to expansion and exaltation, as the human mind advances in knowledge and virtue. That it has often been simulated by heartless shylocks and reckless charlatans, only proclaims its worth. A godless ambition and a lust for gold, have each pushed their adventures even to a more perilous extreme, and maintained their pretensions under the lash of conscience and the denunciations of Heaven. While a misguided and illusory philosophy has sometimes presumptuously thrust its form where "an arch-angel trembling, might fear to tread." The noblest themes have been perverted, the most benevolent enterprizes paralized, and the holiest heights invaded, by the selfish, the sordid, and the profane. Christianity, herself, has had her counterfeits, as well as her avowed assailants, and even false claimants to the Messiahship have challenged the faith of mankind. Theudas drew 400 followers after him, and Judas of Gallilee, "in the days of taxing," led off a giddy and credulous crowd. But both—with their infatuated devotees—met an early and a melancholy fate.

Men will occasionally exemplify the moral of the fable, and don the plumage of the peacock, at the hazard of being plucked bare, and exposing the native homeliness of the jackdaw. Nay, when a prominent interest is likely to suffer, and a favorite craft is in danger, even from the prevalence of *Truth*, they sometimes suddenly become vociferously patriotic or supremely devotional, and the welken rings with the cry "Great is Diana of the Ephesians, whom all Asia and the world worshippeth." But, gentleman, let us magnanimously tolerate these humiliating exhibitions of degraded or duped humanity, nor attempt a Quixotic assault upon every blustering windmill that intercepts our path to usefulness and distinction. Let us return not evil for evil, nor railing for railing. The enviable status of our profession has little to fear from the school-boy squibs that occasionally annoy the highway to greatness; or from the more noisy pyrotechnics of gaseous empirics, which provoke a momentary gaze, and then leave their harmless smoke to be dissipated by the winds of heaven. Still less has its elevation and dignity to apprehend from the *putrid* and *offensive* missiles, which occasionally fly from filthy fingers, to fall far short of their malignant aim, and waste their stench upon the vacant air. Scientific medicine towers its crest and tarnishes its honor, by condescending to encounter every-day humbugs, upon the dusty arena of strife where fistic heroes vapor. Lofty character stoops not to pigmy contests. Swift's Gulliva could but smile at the vindictive outbursts of the Lilliputian miscreants, whose harmless shafts but titilated the surface on which they fell. No, gentlemen, we have nobler game to provoke our ambition. Like the veteran border hunter, we should rather reserve our fire for bears, or buffaloes, than waste our ammunition upon *owls* and *skunks*, lest, forsooth, only feathers and fetor be the bootless reward of our skill.

We are engaged in an honorable work; our reputation is to be built up, not from the "disjecta membra" of a shapeless and dilapidated Empiricism, but independent of its success and in the face of its most imposing pretentions. No high-minded and skillful mechanic traverses the country, far and near, to hunt up crazy cottages or rickety mansions, crooked porticos or clumsy columns, to present them in offensive and ridiculous contrast with his own faultless architecture. No, he magnanimously leaves these exponents of ignorance and awkwardness to meet the just censure of an enlightened popular taste; proceeds to erect his own stately structure, springs its arches, stretches its entablature and lifts its colonade, and by this patent exhibition of his architectual merits, *commands* the patronage for which he will neither condescend to beg nor trick.

Experience and honor, therefore, both counsel us to let professional ignorance alone, even when cozily nestling in the lap of a credulous community, or fostered by the hand of legislative patronage. For the generous representations of the people, while they frequently, and very properly, lend their prestige and their aid to the advancement of infant enterprizes and laudable associations, likely to promote the public good, are sometimes unwittingly committed to the support of pretentions of more than questionable propriety.

At one time, the world had run mad upon the subject of *a cure for madness*, supposed to have been discovered in Europe; and crowned heads and Republics passed laws to preserve, from destruction, the wonderful plant whose virtues were said to disarm Hydrophobia of its horrors. Even the Pennsylvania Legislature appointed a grave committee to receive the disclosure at the hands of Kettering, a German empiric; when lo! the *Pimpirnell*, or common *chick-weed*, was announced as the infallible antidote. The secret was out, the bubble burst, and the charm was lost. The New York Legislature, too, manifested equal sagacity, and gave $1000 for another hopeful remedy for canine madness. Behold the sage prescription, viz:

"1 oz. of the jaw-bone of a *dog*, burned and pounded to dust.

1 False tongue of a *newly foaled colt*, dried and pulverized.

1 ℈. of verdegris from old copper, which has lain under moist earth. *The coppers or pennies of George I. or II. are the best!*"

But there may be a plausible and yet false philosophy which demands refutation before an enlightened public. In this age of progress, gentlemen, our profession is amplifying her resources, studying more profoundly the human organization, and making numerous and valuable additions to the stock of her remedial agents. And yet there are *now*, as there have ever been in past centuries, some monocular dogmatists who can see but *one remedy*, or *one class* of remedies, amid the vast range of the Materia Medica, and authoritatively exclude all others from the treatment of disease. We are even now gravely told that only *vegetable* remedies can safely be administered to regulate derangement of the animal economy—that all *mineral* products are inapplicable, and even dangerous. One of the most popular, and therefore, one of the most formidable objections urged against the regular practice of the profession, is based upon this unsustained and unwarranted assumption. Let us submit, then, this false and unphilosophical conceit, to the test of scientific scrutiny, and practical experience.

Man is an epitomized concentration of the great organic laws which control the *three kingdoms* of Nature. He has been appropriately called, therefore, a "*Microcosm*," (i. e. a "little world.") He consists of *solids*, *liquids*, and *gases*. He embraces in his organization, elements common to the *mineral*, *vegetable*, and *animal* kingdoms. He has, too, in his *own person*, every characteristic property, General and Secondary, assigned to matter.

1st, Its *general* properties, or those without which matter cannot exist, viz:

*Extension*, or the capability of occupying some place in space, and involving the ideas of *form*, length, breath, and thickness;

*Impenetrability*—the property which prevent any other particle of matter from occupying the same place at the same time;

*Mobility*—the susceptibility of motion among the molecules of bodies, and best illustrated in the fluids of the animal body;

*Extreme Divisibility*, or the quality of being separable into minute, and even invisible corpuscles or particles, exemplified in the human organism by the red blood-cells, from 3000 to 6000 of whose diameters are required to make one inch. And still more expressively by the one part of Hydrated Per-oxide of Iron, distributed through 2000 parts of arterial blood, or the 2 parts of Tri-basic Phosphate of Soda, diffused through 10,000 of the same fluid;

*Gravitation*, that force by which all matter is attached to all other matter in the natural universe, and which, on our planet, causes all bodies to tend towards the earth's center;

*Porosity*, the condition of interstitial spaces among the particles of matter;

And *Indestructibility*, or the incapability of being annihilated, save by the power which created it.

2d, Its *Secondary* properties, the presence of *either* of which would identify him with the inorganic material world around him, while in his wonderful organism, we may trace them *all*, e. g.:

*Opacity* and *Transparency*, *Softness* and *Hardness*, *Elasticity* and *Inelasticity*, *Density* and *Rarity*, *Brittleness* and *Toughness*, *Coloration* and *Decoloration*, *Solidity*, *Liquidity*, *Gaseity*, &c. Our time will not allow us to supply in detail specific illustrations of these properties, but any competent Anatomist and Physiologist will not fail to recognize their individual presence in the structure, functions, and phenomena of the animal economy.

The *Six* forms of *Attraction*, too, which govern the relative action of material molecules *beyond* the pale of life, are all now known to exhibit their characteristic results *within* that highly organized and limited circle, viz: Gravitation and Cohesion, as well as Chemical, Capillary, Electrical, and Magnetic Attractions. Aye, *Magnetic* Attraction is neither to be overlooked nor repudiated. Its presence might be inferred, whether the present state of chemico-physical science could illustrate its action or not, for all the magnetic phenomena of our globe are now known to depend upon the presence and power of that invisible, ubiquitous and subtile fluid, which under *one* form of manifestation we call "*Light*," in *another*, "Caloric," in another, "Galvanism," and again, in *another*, "Electricity."

Now we prove the existence of these several phases in the animal body, and being all dependent upon an identity of origin, we may philosophically conclude, that the capabilities for the production of magnetic phenomena, are there also. But Science has already disclosed more than this. Recent discoveries by Prof. Faraday, and others, make it probable that every substance in the terrestrial universe, is either *para-magnetic*, i. e., exhibiting polarity on the axial line, between the two poles of an electro-magnet, or *dia-magnetic*—standing at right angles to the magnetic axis. Every organ and tissue of the human organization, therefore, must be subject to the exactions of this illemitable law.

The oxygen and the iron of the blood are now known to be actively magnetic—the latter being the finest type of its class, while their compounds are found to be in the same state. On the contrary, Sodium, Phosphorus, Sulphur, Chlorine and Hydrogen, with their compounds, including Hydro-chloric Acid and Water—all of which are found in the solids or fluids of the human body—are in the opposite, or dia-magnetic state.

Indeed, in the experiment of Prevost, reported by De La Rive to the French Academy of Science, a steel needle was magnetized by laying it in the equatorial position, over the crural nerve, and irritating the spinal marrow, so as to intensify the neuro-electric current, whose axial line of movement was thus, at an angle of 90 deg. with the needle.

The whole train, then, of what are denominated Imponderable Agents, i. e., Light, Heat, Electricity, and Magnetism, have an accredited reign and wide dominion in this flexile system, while every *department*, into which philosophers have classified the laws of matter, whether *physical* or chemical, in order to facilitate their study, will find a characteristic exemplification here.

1st, *Physics* proper.

*Statics*, or counterpoising forces, producing *rest;* as witnessed in the counteraction of an equilateral atmospheric pressure of 16 tons, upon the surface of a middle-sized man, insensibly effected by the elasticity of the fluids and tissues under the control of functional action, and resulting in a happy and exhilerating equilibrium, which leaves us unconscious of the action of the antagonizing forces producing them.

*Dynamics*, or the philosophy of matter *in motion*, as seen in the beautiful play of the mechanical forces in graceful and easy locomotion:—embracing

*Hydronamics*, with its subordinate division of *Hydrostatics*, or the pressure of fluids at rest, and *Hydraulics*, or their laws of passage along tubes, as exemplified in the pulmonary and systemic circulations.

*Pneumatics*, or the science of elastic fluids, is finely developed, both in its chemical and mechanical aspects, in the normal process of *inspiration* and *expiration*, the oxidation of the blood, and the elimination of carbonic acid gas and watery vapor, from the pulmonary exhalents.

*Optics*, as manifested in the function of the Retina and laws of vision, and

*Acoustics*, in the auditory apparatus and the phenomena of sound.

2d, *Chemistry.*

The vigorous and effective affinities which distinguish this department of Physics, begin their play in embryo, continue it through uterine life, and widen their field of action when the extruded fœtus enters upon its new theater of independent existence in the external world. They preside over the whole physical economy, and regulate the performance of every function, from the primary processes of respiration and alimentation, throughout the oxidation of the blood, the evolution of animal heat, the metamorphosis of the tissues, the decarbonization of the muscles, the deposition of Phosphate of Lime in the bones, the formation of Carbonic Acid, and its expulsion through the superficial emunctories and the air cells of the lungs. The vaporation of transpired serum from the cutaneous surface, &c., &c., continuing their ceaseless and powerful activity until exhausted Nature, disbands, in death, the numerous and obsequious elements, so long held under her vital control, when they are allowed to enter into new combination and take on new forms.

But more than all this: Man is made "a little lower than the Angels." He is a physico-intellectual being, linked to the skies by mental and moral ties. His rational and emotional natures deeply affect, and are, in turn, powerfully affected by his corporeal nature. Over-wrought anxieties, crushed affections, and mental aberration, must, therefore, be treated, not forsooth by *vegetable* remedies *alone*, nor by any class of pharmaceutical preparations merely, but also by moral agencies and social and sympathising appliances, addressed to the sensibilities of his higher nature.

Then this strange and complicated *Microcosm*, when impaired in any of its multitudinous functions, demands, for its restoration to healthy and normal action, judiciously directed remedies, selected from the exhaustless resources of the surrounding *Macrocosm*, or "great world," of which it is the delicate and miniature exponent.

That there is neither philosophically nor practically, any limit to the sphere from which our remedies should be drawn, for the treatment of disease, may be legitimately inferred from the chemical elements employed in our physical constitution.

Once, it is true, Chemistry—when the world was ignorant of her claims, or without sufficient proofs for their enforcement—was excluded from the pale of animal life. Nay, she was even outlawed, as a foot-pad among the Sciences, and her pretensions to a higher reign, subjected her to the scorn and anathema of Philosophy. But thanks to the indomitable and independent spirit of modern research, such distinguished physicists as Chaptal, Raspael, Leibig, Matteucci and Faraday, have recognized the expatriated princess in exile, exchanged her unmerited humiliation for her rightful prerogatives of royalty, and enthroned her within the empire of organic life, while an arrogant Philosophy is now constrained to acknowledge the reign of the sceptered Queen over every element and compound in the natural universe, from the invisible atom of Hydrogen in aqueous vapor, to the gorgeous complexity of planetary organizations, and from the microscopic structure of the infinitisimal monad, up to the wonderful and massive, thinking brain of God's acknowledged vice-gerent upon earth—*lordly* MAN himself.

But why this relentless crusade against inorganic remedies, when it is remembered that the human system is literally built up of external elements, common to the azoic world, and found in rich abundance throughout the solids, fluids and gases of our globe. If, indeed, mineral substances are to be regarded as so deleterious to functional action, and so subversive of animal health, why did the plastic hand of the omniscient Architect construct the primeval model of the human race in its perfectional beauty, out of the inorganic "dust of the ground," or, as we may understand the term, "*dust*," from the "*usus* loquendi" of the orientals, the "*prima* materia," or *elemental matter* of this mundane sphere,

(see Prov. 8th ch., 26th v., and Dr. A. Clark "in loco.") Whatever would be incompatible with the heathful play of its organic laws *now*, must have proved a fatal incorporation in its physical constitution *then*. But, by universal admission, the atomic constitutents of man's natural body, are the same *now*, as in the freshness of their first combination, when God "breathed into his nostrils the breath of life, and he became a living soul;" and amid the perpetual waste of excitement from muscular action, and functional life, it is only by the constant supply of similar particles by the processes of respiration and nutrition, in disease and health, that his period of earthly duration is lengthened out to "three score years and ten."

Let us, then, take a brief though indiscriminate survey of the demands which human Anatomy and Physiology make upon the non-metallic Radicals, Alkalis, Alkaline Earths and Metals, claimed in common with the inanimate mineral kingdom and obtained from it.

*Carbon:* one of the special constituents of Muscle and animal fat, is found in immense quantities in the bituminous and anthracite coal-deposits of our globe, in its limestone strata, and, anon, sparkling in crystaline beauty upon the brow of Royalty, its individual value being sometimes fictitiously estimated at millions of pounds sterling!

*Sulphur:* an ingredient in the human hair, skin, nails, and albuminous matter; and yet a native product in the solfataras, or craters of extinct volcanos, and found extensively mineralized in the form of sulphates and sulphurets, with Iron, Copper, Baryta, Strontia, Antimony, Mercury, &c.

*Phosphorus:* another element of the same class, circulating in the blood, deposited in the brain, and constituting

$\frac{1}{170}$ of the weight of its entire mass selected, as phosphates, from the Mammæ and the Kidneys; and, as Phosphate of Lime, making the great staple of human bones and teeth; and yet, forsooth, a mineral deposit, found in our mountains and plains, in combination with Iron, Lead, Manganese, and Lime.

Then we have *Alkalis*, Soda and Potassa, and the *Alkaline Earths*, Lime and Magnesia, together with the basic metals; of the former, *Sodium* and *Potassium*, and of the latter, *Calcium* and *Magnesium; all* found within the corporeal organization, as circulating salts, or solidified deposits.

The Chloride of Sodium, or domestic salt, whose base is a pure *metal*, is one of the ingredients of healthy blood; is constantly discharged from the cutaneous emunctories by transpiration, and is now an indispensable condiment at every table, and a characteristic test of civilization, thoughout the world. And yet, Nature supplies it liberally from her own Laboratory, in the form of saline lakes, or springs, in the old as well as the new world, producing, by evaporation, from one locality alone 2,000,000 of bushels per annum. While in Siberia, Poland, and Hungary, whole mountains and deep mines of its crystalized masses have been open to the wants of the nations for 600 years.

Need I mention, lastly, that Iron, as purely metalic as that existing in the plow-share or the locomotive, is the great carrier of inspired Oxygen, throughout the systemic circulation, a constant constituent of the Hematosin of the blood, and found in muscle, milk and hair, and yet its native ores are deeply emboweled in the earth, over-hung by the cliffs of the Hartz and the Alleghanies. Indeed, every one of the 15 chemical elements which are found as normal constituents of the human body, belongs equally to the inorganic kingdom, and exists there, either in simple form, or in combination with other elements. Each of these ingredients must, therefore, be supplied in our food and drink, or the animal system deteriorate, and functional disturbances follow.

Why, then, ridiculously prohibit and eschew, by an arbitrary and unphilosophical dogmatism, what Nature claims as her birth-right, and which her hygienic laws perpetually demand? And why insanely insist upon the adoption of a monocratic system of medication in which the employment of vegetable remedies, *alone*, shall be tolerated; and especially when it is remembered that the mere botanical origin of any drug constitutes no sufficient guarantee of its innocuous nature. Nay, the most concentrated and virulent poisons within the whole range of Toxicology, are derived from the "Flora" of different countries; and many of them, without the intervention of pharmaceutical agency, are furnished directly from the roots, barks, juices, leaves, flowers, fruits, seeds, or effluvia of the plants which elaborate them by the functional processes of a vegetable physiology. Mark the following illustrations, which might be indefinitely extended, viz:

*Oxalic Acid:* from the wood-sorrel, (Oxalis Acetosella).

*Narcotine and Morphine:* from Opium—itself a poison.

*Hyoscyamine:* from the Black Henbane.

*Upas Radja:* a dreadful poison, from a climbing shrub in Java—(Strychnos Tieuti).

*Aconite:* one of the most destructive alkaloidal bases known, and obtained from the Wolf's-bane (Aconitum Napellus).

*Strychnine:* a powerful sedative poison, from the Strychnos Nux Vomica.

*Hydrocyanic Acid:* found in the Cherry Laurel (Lauro Cerassus), the Bitter Almond, Peach blossoms, &c.

*Atropia:* from the Deadly Nightshade (Atropa Belladonna), a narcotico-acrid poison.

To these may be added the poisonous principles of the James Town Weed (Datura Stramonium), the American Helebore (Veratrum Viride), and a long list of others. Aye, and last, but not least, in the destructive category, must be enumerated that seductive beverage, our popular Delilah, *Alcohol*, which has sapped the foundation of more constitutions, blasted more hopes, and sent more abandoned paupers, driveling idiots, and raving maniacs, into an untimely and unhallowed grave, than ever fell under the combined Napoleonic thunders of Wagram, Austerlitz, and Marengo.

Human knowledge accumulates through the lapse of years. Ten thousand streamlets, originating from as many fountains, pour their tributary stores into wider channels, and increase the swelling flood, until formidable obstacles, which have heretofore impeded the progress of intelligence and civilization, are successively borne away before the resistless momentum, as the Alpine debris by the careering waters of the Rhone. The student of Nature, therefore, who would reach the highest attainments of human learning, must pass down the stream of history and avail himself of the aggregate contributions of a hundred generations. Not so, however, in regard to the acquisition of *moral* truth, since the Divine code has been completed. To receive it in its fullness, its purity, and its lustre, we must *go back* for eighteen centuries. Then a plenary Revelation bursts upon the world, which has ever since shone with undiminished brightness, and, until the apocalyptic Angel shall sound the doom of the nations, we shall continue to look back upon its unshorn orb for the only true light which marks the path to Heaven.

For the acquirement of all *human* knowledge, then *rational observation* and *patient thought* are the only sure and effective instrumentalities. *Accidents*, it is true, have been the "puncta salientia" of great discoveries; and yet such accidents might transpire for centuries, beyond the reach of sagacious intellect, and the world be none the wiser for their occurrence.

It is only when striking incidents pass in review under the cognizance of *mind*, that great truths are evolved, and facts garnered for future ages.

Carbonic Acid had encircled our planet, and mingled with its elementary gases, in that primeval age when it "was without form and void, and darkness was upon the face of the deep," and the new-born sun had not yet tried his infant beams "athwart the gloom profound." Nay, geologic record, graven with the stylus of passing centuries upon the tablet of the rock forever, has disclosed a superabundance of this compound in the pre-Adamic period of the world's history, polluting the atmosphere by its poisonous intermixture, and making it irrespirable for the higher order of animated forms, and suited only for that palæozoic age, when no air-breathing animals existed; and when Nature, under the stimulus of great heat and moisture, was depositing her carboniferous formations in mountain ranges, covering the more fruitful and boundless plains below with her immense forests, built up, in their ligneous fiber, from the carbon of the decomposed acid, and subsequently overwhelming and inhuming these exhaustless masses by mundane catastrophes, and laying them away in subterranean store-houses, to constitute the extensive *coal-fields* of both hemispheres; while the liberated Oxygen, a depurated atmosphere, and a lower temperature, prepared the way for the age of Mammals, and the reign of Man.

But even in that later era, this gas had been in active play, aiding in the functions of vegetable and animal life, for 5700 years from the Adamic period, and yet its presence and its claims had remained unknown, until the bursting bubbles from a brewer's vat, where some playful urchins were amusing themselves by extinguishing tiny straw matches, caught the eye of a thoughtful philosopher, as he passed the streets of Edinburg. This *incidental* occurrence, which had heretofore been inattentively witnessed by thousands, under the guiding genius of Dr. Black, opened up to the world, the invaluable science of *Pneumatic Chemistry*. The suggestive mind of this great experimenter set Priestly to work, and by the effects of exhaustion upon mice and vegetables within a receiver upon an air-pump, and the subsequent exposure of Per-oxide of Mercury to the sun's heat, under the focus of a condensing lens, he discovered *Oxygen gas*—a philosophical revelation, which, in its consequences, should be regarded, in the language of Dr. Draper, as "little less than gravitation itself."

*Thought*, then, gentlemen—patient, penetrating, powerful thought—has made Medicine what it is, and can alone make her enterprizing votaries what they aspire to be. Nothing less can successfully defend her claims, or elevate her destiny.

It was not *accident*, but *Thought*, which evolved the Novum Organon of Bacon—exploded the Aristotelian Philosophy which had reigned for sixteen-and-a-half centuries, and supplied the world with the Inductive system upon which all true Science now depends for its rational results.

It was *Thought*—noisless but gigantic Thought—blazing in the lights of genius, which marked the accidental fall of an apple in the garden of the great English philosopher and soared them on adventurous wing to the starry heights where Sirius and Aldebaran reveal their perennial glories to the mighty skies—threaded the intricate mazes of stellar and planetary revolutions, and balanced the solar universe in sublime equipoise upon one grand elaborated law, vindicating the majesty of truth, and the power of the human intellect to Angels and to men.

Intense and patient thought, too, tracing mathematical formulæ to their immutable results, revealed to Leverrier in his study, the necessity of some hitherto unknown primitive body in space, to account for the remarkable perturbations in Herschell's moons. Heh ad but to direct Dr. Galle, of the Royal Observatory at Berlin, to point his telescope to that position in the heavens, indicated by his laborious calculations, when lo! on the very *first* evening after the instructions were received, the distant stranger was discovered upon the outskirts of the skies, and *Neptune* was welcomed into the sisterhood of planetary worlds.

Pressing forward under the same intellectual momentum, and braving the discouragements of poverty and obscurity, a poor and dependent French physician has discovered a new planet between Mercury and the Sun, and enrolled his name upon the scroll of fame, so that the detection of L'Escarbeaut's "Vulcan," almost within the blaze of the solar center, is as much the subject of wonder, as that of Leverrier's Neptune.

Thought has been the motive power which has ever borne onward the great minds of our profession to their noblest triumphs. It presided over Harvey's immortal revelation and enabled him to confirm and illustrate it, in his Lectures, for 9 successive years before its publication to the world, while it pioneered the discovery of Jenner, and by the aid of observation and experiment, has stayed the ravages of that fearful scourge of the nation—the Small Pox.

But why need I delay, by further details? The illustrious Temple of Medicine, still in the process of erection, and receiving its solid contribution from the unexhausted veins of thought, must continue to rise, in solemn grandeur, like the stately pile upon Mt. Moriah, without the noise of hammer or of saw, until its beauty and strength shall command the admiration and respect of mankind.

Man is the only earthly creature capable of endless progress in the accumulation of knowledge. All the other members of the Class of Mammifers to which he belongs, even embracing the Quadrumana themselves, stand removed at an immense distance below him—soon reach their acme of improvement, and are then, stationary forever.

Some animals, it is true, besides the monkey family, and mainly birds and quadrupeds, have inferior powers of imitation, by which they may be taught fantastic tricks and curious evolutions, uncommon to the species to which they belong, but they are executed without understanding, taste, or judgment, and can never be communicated to their descendants. The rude articulations of the prating parrot, and the garrulous jack-daw, the clumsy gymnastics of the unwieldy elephant, and the gesticulations of the "dancing dogs," and the "learned antelope," belong to this class. And yet their untutored posterity are found as stupid as their ancestry.

Aye, the lark sang as sweetly when she mounted heavenward, for the first time, upon the morning air of Paradise, as in the nineteenth century, when she carrols her matin melodies over the parks and plains of cultivated England.

Our own mockingbird, too, trilled his jocund song of mimickry as perfectly amid the wild woodlands of an unpeopled continent, 300 years ago, as now, among the pruned groves and pleasure grounds, where science and civilization reign.

Not so with the Order Bimana. *Man*, its only species, is, in one age, a rough and rude warrior, wielding the club and spear in brutal broils, or flinging his nets, and barbing the wild boar. In a succeeding age, his mental developments have elevated and transformed him into an enlightened agriculturist, or skillful artisan, a lofty philosopher, sagacious statesman, or a profound divine. Let the hackneyed but truthful aphorism of the Vis-count of St. Albans, then, ever exert its healthful influence upon the mind of our time-honored profession, viz: "Knowledge is power," and its effects will be felt beyond the limits of the sick room, react favorably upon fame and fortune, and diffuse its grateful and humanizing aroma through all the social and civil relations of life. What were Napoleon's Guards, and Napoleon's Legions, until marshalled and fired by the glowing intellect of the gifted Corsican. What the vapid fumes from the landlady's kettle, or the peasant's pot, until chambered and controlled by the skill and the genius of Fulton. Then, and not till then, under their elastic recoil, a thousand keels cleave the boiling seas, outstripping whole argosies of other days, and ten thousand wheels ring through midlands and mountains, bearing upon their burning axles the tonnage of continents. It is mind, then, at last—trained, expanded, powerful mind—that makes the man, and moves the machinery of nations.

But can this high cultivation be reached, and these ennobling characteristics manifested in the South—the traduced but generous South from which we hail? Who shall successfully deny it? Our extended territory and productive soil, stretching from the alluvium of the seaboard, with its sugar plantations, orange groves, and rice fields, through the primitive and transition regions, with their diversified products of cotton, Indian corn, and other cerials, to the far off secondary in the basin of the Mississippi, with the grapes, and grains, and extensive forests, which adorn the intervening mountain ranges, and their slopes and valleys, afford ample incentives to active industry and talent. While almost all varieties of climate, which the most sensitive and fastidious might desire—from the soft and lambent air of the Atlantic and the Gulf, to the keen, bracing breezes which fan the rugged summits of the Altoonas—are found within her broad domain. But this is not all. Her majestic rivers, her iron mountains, her copper fields, and her rich gold mines, give to the land of the Magnolia and Pine a majesty and a prestige which forecasts a sublime destiny in future ages.

Her governmental ties, her peculiar institutions, and the hostile legislation which, for more than the third of a century, they have provoked, have contributed to repress the growing greatness of her people, and to limit the sphere of their industrial action. But these long continued and tantalizing aggressions, on the part of those who claimed a national confraternity with her, characterised, as they have been, by an unrelenting fanaticism, whose supercilious exactions seemed but to increase with the forbearance of its victims, until a depraved popular opinion found its full embodiment and exponent in the presidential chair, has, at last, aroused her slumbering energies, and evoked her untried powers. Under providential sanction, she has stricken loose the fetters which political tyrants forged, and Freedom has generously bestowed her birth-right upon every recognized citizen and son of her soil. Vigorous intellect is fostered here amid the enlivening diversity of sunshine, and shade, and shower; inherits nobleness from the maternal breast, and it is rationally developed by the educational training of our schools and colleges, and the high moral culture of the Cross. But its utmost capabilities remain yet to be known.

The giant long slept, as if steeped in new wine, while easy conquests lay unsecured within his grasp. The southern heart, however, is now moved by new and stirring impulses and enterprize is reaching to the foundations of society. The ear is every where greeted with the sound of the plowman's whistle and the reaper's song, with the hum of the cotton-gin, and the rush of water-wheels, mingled ever and anon with the blast of furnaces and the thunder of locomotives. While the busy steam-press is disseminating the treasures of literature, and the appliances of science are rapidly advancing the professions and the arts. Indeed, the whole land teems with the elements of power. The salient and plastic mind of our people has begun to seize and appropriate them and some unborn writer shall yet illumine the pages of American history with a glowing chapter upon the grandeur and independence of the South. But accustomed as she has been, from the infancy of our Republic, to lean upon the North, she has heretofore failed properly to appreciate her own intrinsic powers, and exhaustless resources, and has trucklingly yielded to the old regime of training her warm-hearted sons in the inhospitable moral atmosphere of Yale and Cambridge—manufacturing her physicians, tutoring her attorneys, and indoctrinating her divines, amid frozen rivers and ice-bound lakes; and even purchasing her horse-collars and hame-strings, her awl-handles and ax-helves, from beyond the Potomac. By this humiliating submission to Northern claims and manifest want of self-reliance, she but lost that respect which she should proudly have commanded, and provided usurpations which her insulted honor and her chivalry, alike constrained her to resist.

And her let us not be misunderstood.

We would not willingly pluck one plume from the chaplet which encinctures Northern mind, nor invidiously depreciate its meritorious acheivements. But we are neither idiots nor imbeciles, that we should cringe to the arrogance of foreign tutors, or guardians. We live not amid the relaxing heats of the torrid Zone, to sink, over-awed by the glance of power, as the stupid and mindless Sepoy under the rod of Anglo-Saxon rule. Our latitude has been honored above all others in the world's history.

The greatest cities and the most magnificient architecture, the highest civilization and the most powerful empires, the richest agricultural returns and the noblest minds the world ever saw, in past ages, have shone in the same belt of latitute that embraces our Southern States.

There loomed great *Babylon*—Nebuchadnezzar's presumptious boast—with her hundred brazen gates, and her high walls, 60 miles in circuit, and 350 feet high.

There, too, stood proud *Nineveh*, that, in the language of the prophet Nahum, "multiplied her merchants above the stars of heaven," numbering her two-and-a-half millions of inhabitants, and her 1500 towers surmounting her walls and looking down 300 feet from their dizzy summits upon the plains below. Even the fragmentary remains of her ivory palaces and sculptured marbles, recently exhumed from their tombs of centuries by the labors of Ledyard, have excited the wonder of philosophers and will attract the gaze of coming generations.

Palmyra and Persepolis, once the glory of their age, and yet, grand in their ruins, flourished under the same elevation of the sun, as does the land we love.

There, too, towered in unrivaled magnificence the ancient "City of the Sun," with her hundred gates and mighty monuments. In the architectural grandeur, astonishing extent and overwhelming majesty of her palaces of Karnac and Luxor, Thebes has had no equal since the world began.

The temples of Esne and Dendera, too, with their memorable planispheres, and columnar beauties, contribute much to the antiquarian wealth which characterizes this land of wonders where the powerful Pharaohs reigned. Indeed, it is a significant and imposing fact, that amid all the diversified and imposing super and sub-terrene remains of ancient civilization, starting from the almost super-human labors of the templed city of Ellora, with all its spacious courts and colossal statues, hewn out of a hill of solid rock, on the eastern extreme of India, sweeping far westward, among the traceried columns and gorgeous catacombs of Egypt, and bordering in our survey the exhumed cities of Pompei and Herculaneum, passing thence by the pillars of Hercules, and crossing the Northern Atlantic, until lost amid the des-interred architecture of our Southern isthmus, as reported by Humbolt and Stevens, and which was the evidence of a higher civilization than that conceded to the Montezumas and the Aztecs, the subsequent lords of Mexico, not *one* has yet been discovered, worthy of our consideration, under a cold and inhospitable sky or beyond this prolific belt where the sun runs high, and the temperature is mild: unless indeed, such a rude Druidical structure as the stone Henge on

Salisbury plain, in England, and the submerged Round Towers in Ireland, be allowed to claim that distinction. Of these sunken and faded memorials of the past, an English Lyric Poet sweetly sings:

> "On Loch Neagh's banks as the fisherman strays,
> When the clear, cold eve's declining;
> He sees the Round Towers of other days
> In the wave beneath him, shining."

But our time limits a further review in this quarter.

All the great Empires of the world, too, have figured entirely or partially in the same favored Zone. The Babylonian Empire, with its long succession of powerful monarchs; the Persian Empire, under Cyrus the Great, stretching from the Hellespont to the Indus, 2800 miles; and from the Euxine to the sea of Arabia, 200 more; and a large portion of the vast Roman Empire, whose luminous center was the city of palaces, seated upon seven hills, with her 420 Temples, and her 1800 superb mansions, once the mistress of the world, or, in the language of Propertius, "Septem urbs alta jugis, toto quæ præsidet orbi," with her long line of distinguished emperors, and heroes, and statesmen—historians and poets, and orators. The land of the Cæsars, and Pompey, and Cato—of Livy, and Virgil, and Cicero—all verify the postulate assumed. The Grecian Empire, also, whose science, philosophy, literature and martial prowess, long commanded the respect and admiration of surrounding nations, stands upon the same roll. Socrates, and Plato, and Aristotle, adorned the sky of her philosophy. Pericles, Æschines, and Demosthenes, swayed the popular mind by the charms of oratory. Achilles and Agamemnon, Themistocles and Miltiades, led the impetuous hosts of her conquering armies. Heroditus and Xenophon perpetuated her historic memories, while her peerless *Homer* over-swept, on fiery wing, all the cold formularies of legendary rhymers, as well as the hyper-critical punctilios of philological composers, and left an inimitable model of poetic genius for the study of an admiring world, by fusing all the fragmentary subtleties and solid learning of the age into one golden mass of intellectual wealth, and transcendant beauty.

But still more distinguished honors have been providentially assigned to the latitude in which we live. Heaven has honored it as the cradle of our race, and crowned it with the Cross of our redemption. In it, the *first* Adam entered upon life, amid the sweets of paradise, but sinned and doomed his race. And in it, the *second* Adam revealed his unearthly attributes, amid stars and storms and earthquakes, and bore the curse for sinners to win the world to God. Here, too, was the home of Israel's poet-warrior king, and here his royal son, in peaceful splendor, reared upon Mount Moriah, the incomparable Temple, garnished with gold, and radiant with the symbol of the Divine Glory. Bathed in the sunlight of the same mild heaven stands rugged Sinai *now*, to vindicate the historic record of twenty-three hundred years ago, when the descended Godhead, amid smoke, and cloud, and thunder, gave the Moral Law for the government of the nations. While, just across the isthmus, rises the Mount of the Crucifixion, where human pardon, bought in blood, reconciled God to man, and gave the Gospel to dying millions.

Then away with the obsolete and futile, if not invidious charge of southern imbecility, and southern incompetency to achieve the noblest triumps in Science and in Art, in Agriculture and in Morals. Then, gentlemen of the profession, why should we fear to rely upon our native powers, enlightened and enlarged by all the learning and experience of the *past*, and stimulated by all the imperative demands of the

*present.* Let us but scan our motives in the sight of Heaven, and ply our patient energies to the work of progress, and formidable obstacles will sink under our blows like the solid granite in the tunnelled mountain before the resistless onsets of pick-ax and powder.

Then, in future generations, shall our honored fraternity be found enthroned in the confidence of mankind, and their empirical maligners have fled to a more congenial obscurity, before the march of intelligence and the light of truth, as owls and jackalls take covert in their caves and dens, when the sun streaks the East.

In conclusion, gentlemen, and as our earnest aspiration, may the loved land that gave us birth, and whose mellow skies now over-span us, be hailed by our posterity, as it is now hailed by us, an integrant part of this great Confederacy, a blushing beauty among the sisterhood of States, that shall nestle under the Ægis of our incomparable Constitution; with her resources developed, her prowess expanded, and her glory unshorn.

